# Optimization of the Synthesis and Radiolabeling of ZIF-8 Nanoparticles

**DOI:** 10.5812/ijpr-144928

**Published:** 2024-04-08

**Authors:** Mahnaz Ahmadi, Elham Asadian, Mona Mosayebnia, Simin Dadashzadeh, Soraya Shahhosseini, Fateme Ghorbani-Bidkorpeh

**Affiliations:** 1 Medical Nanotechnology and Tissue Engineering Research Center, Shahid Beheshti University of Medical Sciences, Tehran, Iran; 2 Department of Tissue Engineering and Applied Cell Sciences, School of Advanced Technologies in Medicine, Shahid Beheshti University of Medical Sciences, Tehran, Iran; 3 Department of Pharmaceutical Chemistry and Radiopharmacy , School of Pharmacy, Shahid Beheshti University of Medical Sciences, Tehran, Iran; 4 Department of Pharmaceutics and Pharmaceutical Nanotechnology, School of Pharmacy, Shahid Beheshti University of Medical Sciences, Tehran, Iran; 5 Protein Technology Research Center, Shahid Beheshti University of Medical Sciences, Tehran, Iran

**Keywords:** Metal-Organic Frameworks, Synthesis, Radiolabeling, Optimization

## Abstract

**Background:**

Lately, there has been increasing interest in the benefits of metal-organic frameworks, and among them, zeolitic imidazolate frameworks (ZIF - 8) stand out as one of the most commonly employed systems owing to their unique characteristics.

**Objectives:**

Given that properties like particle size play a key role in biomedical applications of nanoparticles, optimizing the synthesis conditions becomes crucial. Additionally, it is essential to label these nanoparticles to track them effectively within the body.

**Methods:**

Zeolitic imidazolate frameworks nanoparticles were synthesized under various conditions, including high and room temperature, using two different solvents: Water and methanol. Modifications were made to the reaction temperature and the ratio of reactants to improve the outcomes. Particle size and size distribution were assessed in all conditions. Additionally, the radiolabeling of nanoparticles was examined using four different methods to identify the method with the highest efficiency and radiochemical purity.

**Results:**

The optimum conditions for ZIF-8 synthesis were determined at 50°C using methanol as the solvent. A reactant weight ratio of 1: 2 (zinc nitrate to 2-methylimidazole) was utilized. The most effective radiolabeling approach involved using tin chloride as a reducing agent, with the reaction mixture maintained at a temperature of 70°C for 30 minutes.

**Conclusions:**

In this study, the optimum conditions were successfully identified for synthesizing and labeling ZIF-8 nanoparticles. These nanoparticles have the potential to serve as effective carriers for diagnostic and therapeutic agents.

## 1. Background

Robson first discovered metal-organic frameworks (MOFs) in 1989 (1), and since then, they have found numerous applications in various fields. These applications include electrochemistry, purification, separation, catalysts, and gas storage (2, 3). Furthermore, in recent years, an emerging area of application for MOFs is in the pharmaceutical and medical fields (4, 5).

Metal-organic frameworks are porous crystalline materials resulting from the connection of metal ions and organic ligands (6). Nano-scaled MOFs (NMOFs) exhibit distinctive properties that render them highly suitable as nanocarriers for drug delivery and bioimaging. Nano-scaled MOFs have demonstrated several advantages including compositional and structural diversity (7), high surface area and porosity, and the ease of surface functionalization (8, 9). Given these desirable features, MOFs have recently garnered significant attention, particularly in the field of biomedicine (10, 11). One of the critical factors in biomedical applications of nanoparticles is their size, which directly impacts their properties such as biodistribution and clearance. Nanoparticles tend to reduce drug delivery effectiveness when their size exceeds 200 nm due to increased toxicity and macrophage-mediated elimination. Conversely, particles smaller than 100 nm exhibit enhanced intra-body transmission and biodistribution within various body sites (12). Research on MOFs indicates that cell uptake and drug release are influenced by particle size (13). Size control of MOFs can be attained by modifying the metal ion and ligand or by adjusting synthesis parameters such as temperature, time, and reactant concentration (14).

Among MOFs, Zn-based MOFs (i.e., zeolitic imidazolate frameworks (ZIFs)) have attracted noteworthy consideration, particularly in the biomedical fields (10, 15). Zeolitic imidazolate frameworks, which consists of zinc metal and 2-methyl imidazole ligand, is extensively utilized due to its inherent characteristics, including low toxicity, exceptional thermal and chemical stability, and extremely high surface area (8, 16).

One of the critical factors in biomedical applications of nanoparticles is their size, which directly impacts their properties such as biodistribution and clearance. Nanoparticles tend to reduce drug delivery effectiveness when their size exceeds 200 nm due to increased toxicity and macrophage-mediated elimination. Conversely, particles smaller than 100 nm exhibit enhanced intra-body transmission and biodistribution within various body sites (12). Research on MOFs indicates that cell uptake and drug release are influenced by particle size (13). Size control of MOFs can be attained by modifying the metal ion and ligand or by adjusting synthesis parameters such as temperature, time, and reactant concentration (14).

Zeolitic imidazolate frameworks nanoparticles find significant application in various imaging techniques for diagnostic purposes (17, 18). Radiolabeling of nanoparticles by a radioisotope (as a marker) can be used to track the nanosystem in the physiological environment, detect various lesions, and investigate their biodistribution, clearance, and pharmacokinetics (19). Labeling nanoparticles in clinical studies can pave the way for diagnosing diseases using various imaging techniques like nuclear medicine imaging and treating diseases, for example, through radionuclide therapy. Moreover, it can offer theranostic nanoparticles for both diagnosing and treating diseases simultaneously (20).

The selection of an appropriate radiolabeling method for nanoparticles is of paramount importance in accurately monitoring their biodistribution within the body.

## 2. Objectives

Given the significance of nanoparticle size and size distribution in biomedical studies, this study focuses on exploring various synthesis conditions to determine the optimal synthesis parameters for ZIF-8 preparation. Additionally, due to the importance of selecting the most suitable radiolabeling method to track nanoparticles in a physiological environment, various radiolabeling techniques were examined to identify the most efficient approach.

## 3. Methods

### 3.1. Materials

Methanol, Zinc nitrate hexahydrate [Zn (NO_3_)_2_.6H_2_O], 2-methylimidazole, Tin (II) chloride dihydrate (SnCl_2_.2H_2_O), and hydrochloric acid were purchased from Sigma-Aldrich. Thin-layer chromatography (TLC) Silica gel (SG) 60 F254 aluminum sheets were obtained from Merck KGaA Ltd., Germany. The [99Mo]/[99mTc] generator was purchased from the Pars-isotope center in Tehran, Iran.

### 3.2. Synthesis of Zeolitic Imidazolate Frameworks Nanoparticles Using a Solvothermal Method

To prepare ZIF-8 nanoparticles using the solvothermal method, a solution was prepared by dissolving 5 g of 2-methylimidazole in 25 mL of methanol. This solution was then added to a separate solution of Zn (NO_3_)_2_.6H_2_O, which was prepared using 2.305 grams of zinc nitrate and 25 mL of methanol. The resulting mixture was transferred into the autoclave chamber and subsequently subjected to high temperatures (100°C) in an oven for 16 hours. Afterward, the mixture inside the autoclave chamber was centrifuged to isolate the resulting solid, which manifested as ZIF-8 particles (21).

### 3.3. Synthesis of Zeolitic Imidazolate Frameworks Nanoparticles in Water at Room Temperature

The synthesis procedure commenced at room temperature using water as the solvent (22). Initially, 1.17 g of Zn (NO_3_)_2_.6H_2_O was dissolved in 8 mL of deionized water, and subsequently, 22.70 g of 2-methylimidazole was dissolved in 80 mL of deionized water. While stirring, the zinc nitrate solution was gradually added to the 2 - methylimidazole solution. The mixture was stirred at room temperature for a few minutes until a cloudy mixture formed. Subsequently, the resulting mixture underwent centrifugation and was subjected to three rounds of washing with deionized water. Finally, it was dried in an oven at 65°C for 12 hours (22).

### 3.4. Synthesis of Zeolitic Imidazolate Frameworks Nanoparticles in Methanol at Room Temperature

Furthermore, ZIF-8 nanoparticles were prepared in methanol solvent. In summary, 1.620 g of 2 -methylimidazole and 1.468 g of Zn (NO_3_)_2_.6H_2_O were separately dissolved in 100 mL of methanol. Then, the solution of 2-methylimidazole was added to the Zn(NO_3_)_2_.6H_2_O solution while stirring, and it remained unchanged for one hour at room temperature. The resulting mixture was then subjected to centrifugation for 10 minutes, and the initial product was washed three times using methanol before being dried in an oven at 70°C for 12 hours (23).

**Figure 1.**
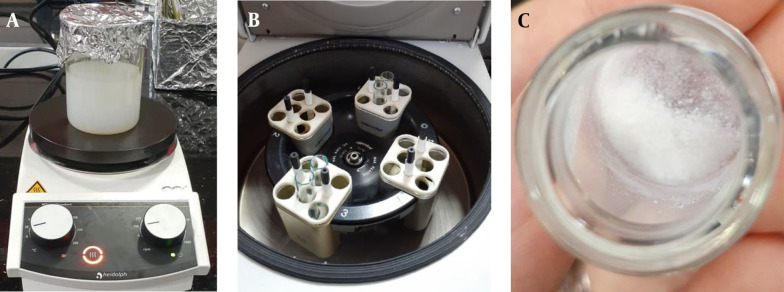
Synthesis steps of zeolitic imidazolate frameworks (ZIF-8) nanoparticles (A, a mixture of reactants; B, centrifugation; C, nanoparticles after drying)

Figure 1 briefly illustrates the synthesis steps of ZIF-8 nanoparticles, including the mixing of reactants (Figure 1A), centrifugation (Figure 1B), and obtaining nanoparticles after drying (Figure 1C). 

### 3.5. Optimization of Synthesis Conditions

The synthesis of nanoparticles was optimized by adjusting various synthesis parameters to achieve the optimal particle size and polydispersity index (PDI). The influence of solvent on nanoparticles was examined in earlier stages. Another parameter that underwent modification was the reaction temperature. The reactant mixture was exposed to temperatures of 30, 50, and 80°C for one hour, and the resulting alterations in particle size and PDI were assessed and compared.

Subsequently, the weight ratio of the reactants was also varied (1: 1, 1: 2, and 2: 1). Finally, the reaction was explored under two conditions, with and without stirring. Particle size and PDI were compared to assess the impact of stirring on the resultant particles.

### 3.6. Radiolabeling of Zeolitic Imidazolate Frameworks with [99mTc]

Different methods were examined to radiolabel ZIF-8 nanoparticles with ^99m^Tc since it has not been conducted before. Four radiolabeling techniques were developed by altering the temperature, time, concentration of nanoparticles, the amount of SnCl_2_, and added radioactivity. The developed methods were compared based on radiochemical purity (%RCP) and radiolabeling efficiency (%RE), and the best technique was selected based on the highest % RCP and % RE.

First, a dispersion of ZIF-8 nanoparticles with a concentration of 1 mg/mL in deionized water was prepared. In the next step, a solution of SnCl_2_ was prepared. To prepare this solution, 10 mg of divalent SnCl_2_ was dissolved in 1 mL of 0.01 M hydrochloric acid (HCl). The acidic solution of HCl must be degassed by nitrogen gas before use. What is needed as a source of technetium from the generator is sodium pertechnetate (Na^99m^TcO_4_), which can be reduced using SnCl_2_. Among the four labeling methods performed, the SnCl_2_ solution was used as a pertechnetate reducer in two methods, and this substance was not used in the other two methods. The details of the four radiolabeling methods are reported in Table 1. A kit (or radiolabeling kit) is defined as a prepared mixture to be combined with a radioisotope.

**Table 1.** The Details of Four Radiolabeling Methods

Kit	T (°C)	T (min)	Concentration of NPs (mg/mL)	Concentration of SnCl_2_ (mg/mL)	Volume of NPs (µL)	Volume of SnCl_2_ (µL)	Added Radioactivity (mCi)
**1**	RT	30	1	10	500	100	6
**2**	70	30	1	10	500	100	6
**3**	70	30	1	-	500	-	6
**4**	RT	30	1	-	500	-	6

Abbreviations: T, temperature; t, time; NPs, nanoparticles; RT, room temperature.

**Figure 2.**
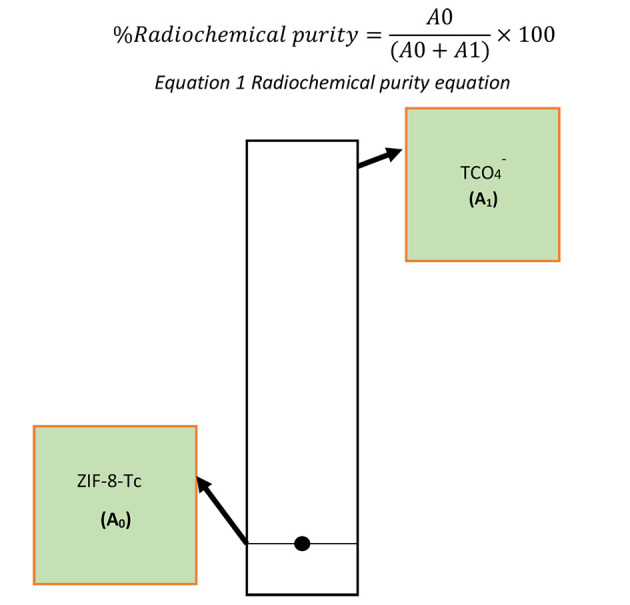
Thin-layer chromatography (TLC) paper in calculating radiochemical purity of radiolabeled zeolitic imidazolate frameworks (ZIF-8)

After 30 minutes, the radiolabeled samples were centrifuged at 10,000 rpm for 15 minutes. The sediment obtained was washed twice and centrifuged again to separate the unreacted pertechnetate from the radiolabeled samples. After centrifugation, the %RE was obtained by dividing "a" by "b" multiplied by 100, where "a" represents the radioactivity of the sediment obtained after centrifugation (labeled nanoparticle activity), and "b" represents the primary radioactivity added to the sample (total activity). Next, TLC was conducted to check the %RCP of each radiolabeled sample according to Figure 2. For this purpose, acetone and ITLC-SG were used as the mobile and stationary phases, respectively. After the mobile phase rose in the TLC paper and the sample moved with the solvent front, the radioactivity value of the upper third and lower third of the paper was measured by the Courimeter device. The %RCP was determined based on the radioactivity of the upper and lower parts of the TLC paper. To calculate the %RCP by TLC, Equation 1 can be used, where A0 represents the activity of the lower part of the TLC paper, and A1 represents the activity of the upper part of the TLC paper.

Moreover, to ensure the retention of technetium within the nanoparticle structure over time and prevent its release, stability assessments were conducted in both PBS (phosphate-buffered saline) buffer and human plasma for up to 24 hours. These investigations involved the utilization of TLC to monitor the amount of released pertechnetate at various intervals within the 24-hour timeframe.

## 4. Results

### 4.1. Synthesis of Zeolitic Imidazolate Frameworks Nanoparticles

Zeolitic imidazolate frameworks nanoparticles were synthesized under various conditions, employing water and methanol solvents at different temperatures. The solvothermal approach, conducted at high temperature and pressure, failed to yield particles with a suitable size and PDI. Consequently, an alternative method was employed at room temperature, utilizing deionized water and methanol solvents. Deionized water resulted in larger particles, prompting the use of methanol as a solvent. Synthesis using methanol at room temperature proved more successful in achieving the desired particle size and PDI. The synthesis was performed at 30, 50, and 80°C using methanol. The best results, in terms of particle size and PDI, were observed at 50°C.

**Figure 3.**
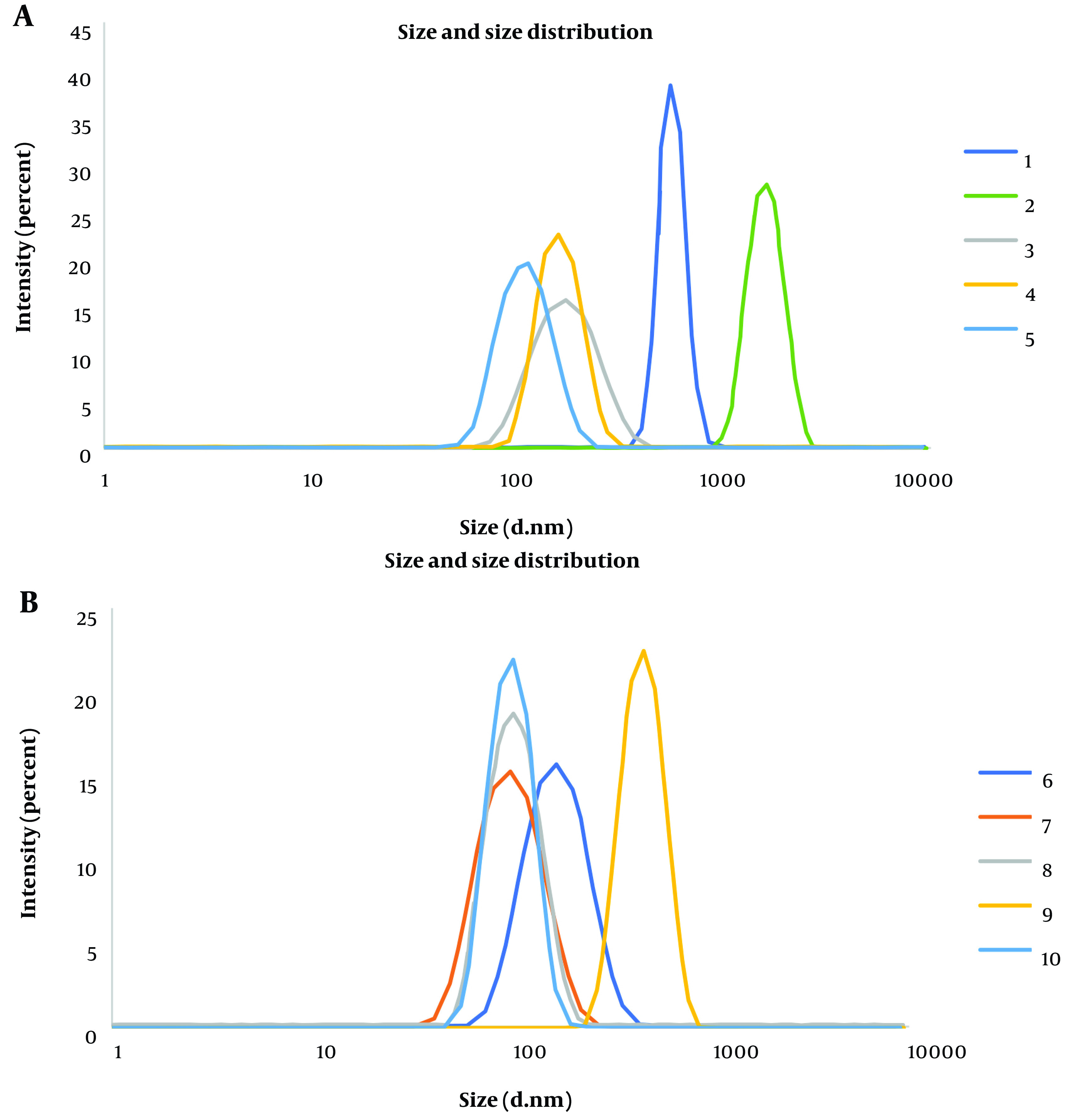
Size and size distribution of nanoparticles obtained from 1 - 5 synthesis methods (above), and 6 - 10 synthesis methods (below)

Once the desired temperature of 50°C was established, synthesis was carried out at this temperature while adjusting the weight ratio of the reactants. Three different weight ratios of zinc nitrate to 2-methylimidazole were employed, including 1: 2, 1: 1, and 2: 1. The weight ratio of 1: 2 (zinc nitrate to 2-methylimidazole) resulted in the optimum size and PDI of ZIF-8 nanoparticles. In this scenario, the amount of zinc nitrate remained constant based on the initial synthesis method, while twice the amount of 2-methylimidazole was used. Furthermore, it was observed that allowing the mixture to stand without stirring resulted in smaller particles. Table 2 demonstrates particle size and PDI values in various synthesis conditions for three repetitions of synthesis. Additionally, the size and size distribution of ZIF-8 nanoparticles obtained from the DLS technique were illustrated in Figure 3. 

**Table 2.** Particle Size and PDI in Various Synthesis Conditions (n = 3) ^a^

Method	Temperature (°C)	Reaction Time (h)	Solvent	Reactant Amount	Size (nm)	PDI (nm)
**1**	100	16	Methanol	5 g 2-methyl imidazole; 2.305 g zinc nitrate; 50 mL methanol	1050 ± 28.3	0.725 ± 0.05
**2**	RT	0.25	Deionized water	22.7 g 2-methyl imidazole; 1.17 g zinc nitrate; 88 mL DI water with stirring	1983 ± 27.73	0.362 ± 0.23
**3**	RT	1	Methanol	1.125 g 2-methyl imidazole; 1.101 g zinc nitrate; 150 mL methanol	185.14 ± 3.045	0.299 ± 0.049
**4**	30	1	Methanol	1.125 g 2-methyl imidazole; 1.101 g zinc nitrate; 150 mL methanol	180.5 ± 4.83	0.27 ± 0.032
**5**	50	1	Methanol	1.125 g 2-methyl imidazole; 1.101 g zinc nitrate; 150 mL methanol	117.83 ± 4.15	0.214 ± 0.027
**6**	80	1	Methanol	1.125 g 2-methyl imidazole; 1.101 g zinc nitrate; 150 mL methanol	192 ± 8.09	0.285 ± 0.023
**7**	50	1	Methanol	1.101 g zinc nitrate; 2.202 g 2-methyl imidazole; (1:2 ratio); 150 mL methanol	96.3 ± 1.25	0.16 ± 0.03
**8**	50	1	Methanol	1.101 g zinc nitrate; 1.101 g 2-methyl imidazole (1:1 ratio); 150 mL methanol	118.75 ± 4.18	0.28 ± 0.017
**9**	50	1	Methanol	2.250 g zinc nitrate; 1.125 g 2-methyl imidazole (2:1 ratio); 150 mL methanol	685.66 ± 21.19	0.486 ± 0.01
**10**	50	1	Methanol	1.101 g zinc nitrate; 2.202 g 2-methyl imidazole (1:2 ratio); 150 mL methanol (With stirring)	145.3 ± 4.85	0.439 ± 0.017

Abbreviations: RT, room temperature; SD, standard deviation; PDI, Polydispersity Index.

^a^ Values are expressed as mean ± SD.

### 4.2. Radiolabeling of Zeolitic Imidazolate Frameworks Nanoparticles

The radiolabeling of ZIF-8 was performed in four different ways, and %RE and %RCP were calculated to determine the best method. As shown in Table 3, the second method provided the highest %RE and %RCP for radiolabeling ZIF-8 nanoparticles (> 95%). Therefore, a suitable method for the radiolabeling of ZIF-8 nanoparticles is to use SnCl_2_ as a pertechnetate reducer and to carry out the radiolabeling process at 70°C for 30 minutes.

Following the successful radiolabeling of nanoparticles using an effective method to achieve high efficiency and purity, stability assessments were performed on the labeled nanoparticles for up to 24 hours in PBS buffer and human plasma, separately. The outcomes from these studies demonstrated the excellent stability of the labeled ZIF-8 nanoparticles, with less than ten percent of technetium being released from the nanoparticle structure for up to 24 hours.

**Table 3.** Radiochemical Purity and Radiolabeling Efficiency for Four Radiolabeling Methods (n = 3) ^a^

Kit	RE	RCP
**1**	47.66 ± 3.51	61.35 ± 4.12
**2**	96.79 ± 1.78	97.43 ± 1.15
**3**	0	0
**4**	10.04 ± 2.18	18.56 ± 4.17

^a^ Values are expressed as No. (%) or mean ± SD.

## 5. Discussion

Zeolitic imidazolate frameworks nanoparticles were synthesized under ten different conditions, utilizing various solvents and ratios of reactants at different temperatures. As indicated in the results section, the most favorable outcomes were achieved when methanol solvent was employed, the temperature was approximately 50°C, and the ratio of 2-methylimidazole to zinc nitrate was maintained at 2: 1. Additionally, conducting the reaction in a stationary state without stirring resulted in better particle size.

Previously, optimization of ZIF-8 nanoparticle synthesis has focused primarily on the synthesis method. For instance, Lee et al. explored various techniques, including solvothermal, microwave-assisted, sonochemical, mechanochemical, dry-gel, and microfluidic methods. They compared particle sizes obtained from different routes and found that particles synthesized via dry-gel and sonochemical methods were significantly smaller than those from other techniques. However, factors such as temperature and reactant ratios were not examined in their study (24). In this study, alongside the synthesis method, the impact of synthesis conditions, including temperature, time, and reactant ratios, on nanoparticle output was also investigated.

In this study, the solvothermal method was initially chosen due to its widespread use in MOF synthesis, allowing for precise control of particle properties by adjusting reaction conditions (25, 26). However, in this specific investigation, the solvothermal method yielded larger particles. Previous research by Usman et al. also noted that the solvothermal method alone typically produces particles ranging in size from several micrometers, necessitating additional techniques such as microwave-assisted methods (27). Moreover, the choice of solvent significantly influences the characteristics of ZIF-8 nanoparticles (28). Several studies have recommended methanol due to its lower risks compared to other solvents, and its effectiveness in achieving suitable particle sizes for ZIF-8 nanoparticles has been demonstrated (29). Additionally, the reaction temperature has been found to impact the size of ZIF-8 nanoparticles (30). Following synthesis, ZIF-8 nanoparticles were radiolabeled using the ^99m^Tc radioisotope in four different methods, among which the method employing SnCl_2_ as a reducing agent and maintaining the reaction mixture at 70°C for 30 minutes demonstrated the highest %RE and %RCP.

The ^99m^Tc radioisotope was chosen as the primary option for radiolabeling ZIF-8 nanoparticles due to its availability and affordability, enabling efficient nanoparticle labeling. The radiolabeling methods were selected based on the expertise of radiopharmacy specialists, utilizing trial and error approaches, and informed by findings from Alberto's study (31). The radiolabeling of ZIF-8 nanoparticles with ^99m^Tc likely occurs through the formation of a dative bond between Tc and the nitrogen of the imidazole ring in the ZIF-8 structure.

Certain aspects of ZIF-8 nanoparticle studies, such as characterization results and stability assessments, are comprehensively documented in another publication by the authors' team (8). However, the present study focuses on optimizing the synthesis and radiolabeling processes, pioneering investigations into the effects of temperature, reactant quantity, and reaction time on achieving the ideal size and PDI of ZIF-8 nanoparticles. Additionally, successful radiolabeling with ^99m^Tc was achieved for the first time with remarkable efficiency, purity, and stability. These labeled nanoparticles hold promising potential for future applications in drug delivery and imaging research.

### 5.1. Conclusions

Synthesis parameters, including temperature, reactant ratios, reaction time, and solvent choice, can profoundly impact the size of ZIF-8 nanoparticles, a critical determinant of their biological behavior. Radiolabeling ZIF-8 nanoparticles with technetium-99m can be accomplished with high efficiency and purity, facilitating their tracking within the body for a specified duration. To optimize the performance of nanoparticles, especially those designated for drug delivery and imaging within the body's physiological environment, meticulous control over the synthesis and labeling processes is essential.

## Data Availability

Data will be made available on request.

## References

[1] Hoskins BF, Robson R (2002). Infinite polymeric frameworks consisting of three dimensionally linked rod-like segments.. J Am Chem Soc..

[2] Meek ST, Greathouse JA, Allendorf MD (2010). Metal‐Organic Frameworks: A Rapidly Growing Class of Versatile Nanoporous Materials.. Adv Mater..

[3] Pettinari C, Marchetti F, Mosca N, Tosi G, Drozdov A (2017). Application of metal − organic frameworks.. Polym Int..

[4] Mendes RF, Figueira F, Leite JP, Gales L, Almeida Paz FA (2020). Metal-organic frameworks: a future toolbox for biomedicine?. Chem Soc Rev..

[5] Moharramnejad M, Ehsani A, Shahi M, Gharanli S, Saremi H, Malekshah Eshaghi R (2023). MOF as nanoscale drug delivery devices: Synthesis and recent progress in biomedical applications.. J Drug Deliv Sci Technol..

[6] Liu Y, Zhao Y, Chen X (2019). Bioengineering of Metal-organic Frameworks for Nanomedicine.. Theranostics..

[7] Cai W, Chu CC, Liu G, Wang YX (2015). Metal-Organic Framework-Based Nanomedicine Platforms for Drug Delivery and Molecular Imaging.. Small..

[8] Ahmadi M, Khoramjouy M, Dadashzadeh S, Asadian E, Mosayebnia M, Geramifar P (2023). Pharmacokinetics and biodistribution studies of [99mTc]-Labeled ZIF-8 nanoparticles to pave the way for image-guided drug delivery and theranostics.. J Drug Deliv Sci Technol..

[9] Pouyanfar N, Ahmadi M, Ayyoubzadeh SM, Ghorbani-Bidkorpeh F (2024). Drug delivery system tailoring via metal-organic framework property prediction using machine learning: A disregarded approach.. Mater Today Commun..

[10] Zhang S, Pei X, Gao H, Chen S, Wang J (2020). Metal-organic framework-based nanomaterials for biomedical applications.. Chin Chem Lett..

[11] Gatou MA, Vagena IA, Lagopati N, Pippa N, Gazouli M, Pavlatou EA (2023). Functional MOF-Based Materials for Environmental and Biomedical Applications: A Critical Review.. Nanomater (Basel)..

[12] Gao X, Cui R, Ji G, Liu Z (2018). Size and surface controllable metal-organic frameworks (MOFs) for fluorescence imaging and cancer therapy.. Nanoscale..

[13] Li S, Tan L, Meng X (2020). Nanoscale Metal‐Organic Frameworks: Synthesis, Biocompatibility, Imaging Applications, and Thermal and Dynamic Therapy of Tumors.. Adv Funct Mater..

[14] Sabzehmeidani MM, Kazemzad M (2023). Recent advances in surface-mounted metal-organic framework thin film coatings for biomaterials and medical applications: a review.. Biomater Res..

[15] Cai W, Zhang W, Chen Z (2023). Magnetic Fe(3)O(4)@ZIF-8 nanoparticles as a drug release vehicle: pH-sensitive release of norfloxacin and its antibacterial activity.. Colloids Surf B Biointerfaces..

[16] Assila O, Vilaca N, Bertao AR, Fonseca AM, Parpot P, Soares O (2023). Optimization of iron-ZIF-8 catalysts for degradation of tartrazine in water by Fenton-like reaction.. Chemosphere..

[17] Zhao Y, Pang B, Detering L, Luehmann H, Yang M, Black K (2018). Melanocortin 1 Receptor Targeted Imaging of Melanoma With Gold Nanocages and Positron Emission Tomography.. Mol Imaging..

[18] Wang D, Wu Q, Ren X, Niu M, Ren J, Meng X (2024). Tunable Zeolitic Imidazolate Framework-8 Nanoparticles for Biomedical Applications.. Small Methods..

[19] Lee DS (2018). Radionanomedicine: Combined Nuclear and Nanomedicine..

[20] Enrique MA, Mariana OR, Mirshojaei SF, Ahmadi A (2015). Multifunctional radiolabeled nanoparticles: strategies and novel classification of radiopharmaceuticals for cancer treatment.. J Drug Target..

[21] Mendes PA, Rodrigues AE, Horcajada P, Serre C, Silva JA (2014). Single and multicomponent adsorption of hexane isomers in the microporous ZIF-8.. Microporous Mesoporous Mater..

[22] Pan Y, Liu Y, Zeng G, Zhao L, Lai Z (2011). Rapid synthesis of zeolitic imidazolate framework-8 (ZIF-8) nanocrystals in an aqueous system.. Chem Commun (Camb)..

[23] Sánchez-Laínez J, Zornoza B, Friebe S, Caro J, Cao S, Sabetghadam A (2016). Influence of ZIF-8 particle size in the performance of polybenzimidazole mixed matrix membranes for pre-combustion CO2 capture and its validation through interlaboratory test.. J Member Sci..

[24] Lee Y, Jang M, Cho H, Kwon H, Kim S, Ahn W (2015). ZIF-8: A comparison of synthesis methods.. Chem Eng J..

[25] Subudhi S, Rath D, Parida KM (2018). A mechanistic approach towards the photocatalytic organic transformations over functionalised metal organic frameworks: a review.. Catal Sci Technol..

[26] Ahmadi M, Ebrahimnia M, Shahbazi MA, Keçili R, Ghorbani-Bidkorbeh F (2022). Microporous metal–organic frameworks: Synthesis and applications.. J Ind Eng Chem..

[27] Usman KAS, Maina JW, Seyedin S, Conato MT, Payawan LM, Dumée LF (2020). Downsizing metal–organic frameworks by bottom-up and top-down methods.. NPG Asia Mater..

[28] Santoso E, Ediati R, Istiqomah Z, Sulistiono DO, Nugraha RE, Kusumawati Y (2021). Facile synthesis of ZIF-8 nanoparticles using polar acetic acid solvent for enhanced adsorption of methylene blue.. Microporous Mesoporous Mater..

[29] Garcia-Palacin M, Martinez JI, Paseta L, Deacon A, Johnson T, Malankowska M (2020). Sized-Controlled ZIF-8 Nanoparticle Synthesis from Recycled Mother Liquors: Environmental Impact Assessment.. ACS Sustain Chem Eng..

[30] Chen Y, Tang S (2019). Solvothermal synthesis of porous hydrangea-like zeolitic imidazole framework-8 (ZIF-8) crystals.. J Solid State Chem..

[31] Alberto R, Krause W (2005). New Organometallic Technetium Complexes for Radiopharmaceutical Imaging.. Contrast Agents III: Radiopharmaceuticals – From Diagnostics to Therapeutics..

